# Advancing cardiovascular tissue engineering

**DOI:** 10.12688/f1000research.8237.1

**Published:** 2016-05-31

**Authors:** George A. Truskey

**Affiliations:** 1 Department of Biochemical Engineering, Duke University, Durham, NC, USA

**Keywords:** Tissue engineering, cardiovascular, iPSCs

## Abstract

Cardiovascular tissue engineering offers the promise of biologically based repair of injured and damaged blood vessels, valves, and cardiac tissue. Major advances in cardiovascular tissue engineering over the past few years involve improved methods to promote the establishment and differentiation of induced pluripotent stem cells (iPSCs), scaffolds from decellularized tissue that may produce more highly differentiated tissues and advance clinical translation, improved methods to promote vascularization, and novel *in vitro* microphysiological systems to model normal and diseased tissue function. iPSC technology holds great promise, but robust methods are needed to further promote differentiation. Differentiation can be further enhanced with chemical, electrical, or mechanical stimuli.

## Introduction

Tissue engineering involves the development of functional replacements for damaged tissues or organs ( http://www.nibib.nih.gov/science-education/science-topics/tissue-engineering-and-regenerative-medicine). A common approach to produce engineered tissues is to add cells to a natural or synthetic extracellular matrix, which provides mechanical support and biochemical cues. Scaffold-free tissues are prepared by growing cells on thermally responsive polymers to facilitate the cell monolayers that form and then adding layers together or rolling the sheets. By addition of small molecules that activate specific differentiation pathways, three-dimensional organoids can be derived from human pluripotent stem cells ^1^. The engineered tissue may be prepared wholly or partially before implantation to activate and localize the body’s regenerative capacity to populate the implanted scaffold. Tissue engineering is a subset of the broader field of regenerative medicine, which seeks to repair or replace damaged organs. This could occur by direct injection of cells or modifying cellular processes to initiate repair and regrowth. In spite of significant research advances and insightful application of developmental cell biology, cell mechanics, and biomaterials, few products have emerged from these efforts to date, pointing to the challenges to develop truly functional tissues.

Key design goals to produce functional tissues *in vitro* are to reproduce the tissue structure and cell density *in vivo*, identify suitable sources of cells, promote growth and differentiation of cells, design a construct that reproduces the extracellular matrix with the appropriate molecular cues and suitable mechanical properties, and create a vasculature within the construct to enable oxygenation and integration with host vasculature after implantation.

**Figure 1.**
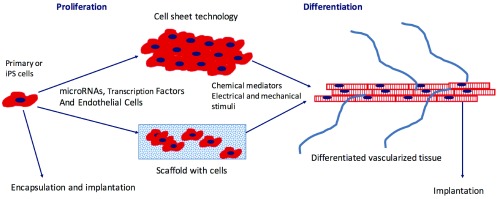
Schematic of processes to produce cell-based engineered cardiac or vascular tissue. Primary cells or induced pluripotent stem (iPS) cells undergo a period of proliferation prior to seeding into a three-dimensional scaffold or are grown as sheets on a polymer whose conformation changes in response to temperature or other stimuli, enabling detachment on the sheet. During the proliferation phase, microRNAs or transcription factors may be added to the cells to promote subsequent differentiation. To promote further differentiation, small molecules are added and/or cells are exposed to electrical or mechanical stimuli. Endothelial cells added to the tissue during formation promote vascular network formation. After the cells have reached a certain level of maturity, the engineered tissue is implanted and the host blood supply connects with the vascular network promoted by endothelial cells. Alternatively, cells may be encapsulated in a biodegradable polymer and implanted.

Several strategies that have emerged to address these challenges include the use of induced pluripotent stem cells (iPSCs) or embryonic stem cells (ESCs) that can differentiate into the cells of interest, the reprogramming of primary cells to the cell type of interest, various ways to engineer the structural support for the cells to mimic the extracellular matrix, and efforts to promote vascular network formation ( Figure 1). Over the past few years, a new use of tissue engineering has emerged in which microscale human tissue-engineered systems or microphysiological systems are used to model normal and disease states *in vitro* and assess drug responses.

Recent advances in tissue engineering over the past three years were recently summarized in two reviews ^2, 3^. Given the breadth of tissue engineering research (13,661 publications since 1 January 2014 reported on Google Scholar), we focus this summary of recent work on cardiovascular tissue engineering as a way to demonstrate how new research results have addressed the key design challenges. Cardiovascular tissue engineering is a vibrant area of research, and applications in the cardiovascular system include cardiac patches, engineered blood vessels and heart valves, and vascular networks.

## Scaffolds

Scaffold materials should match the mechanical properties of the tissue and undergo degradation or be integrated into the tissue, allowing the natural extracellular matrix to replace the original structural support. Materials used in cardiovascular tissue engineering include degradable polymers, such as polyglycolic acid ^4^ and polylactic acid, as well as biological hydrogels, such as collagen ^5^, fibrin ^6^, and modified hyaluronic acid ^7^. These scaffold materials can be modified by the addition of cell adhesion domains or sites susceptible to cleavage by matrix metalloproteinases to facilitate cell attachment and migration. Alternatively, sheets of cells can be prepared and then fabricated into different configurations such as tubes or patches. Such structures have high mechanical strength and function well *in vivo* ^8^. This approach has the advantage of not needing any synthetic polymers.

A scaffold-free cardiac patch consisting of three layers of rat cardiomyocytes was successfully engrafted onto heart tissue by overlaying the patch over a vascular supply, enhancing the ability of endothelial cells (ECs) in the patch to form a functional tubular vascular network connected to the host blood supply ^9^. Recently, the development of a porous patch with an electroactive polypyrrole incorporating electronics for sensing and stimulating electrophysiological activity and release of various biological molecules offers a new level of control of cardiac patches while permitting incorporation of cells attached to a bioactive scaffold ^10^.

While tissue-engineered blood vessels (TEBVs) constructed from natural matrix components such as collagen ^11, 12^ and fibrin ^13, 14^ have traditionally exhibited poor mechanical strength, plastic compression of collagen gels embedded with smooth muscle cells (SMCs) increases the collagen fiber density and yields rapidly producible tubular structures with high mechanical strength ^15^. By plastic compression of collagen, TEBVs with burst pressures exceeding 1600 mmHg can be prepared in a few hours ^5^. After one week of perfusion at physiological shear stresses, the medial cells exhibited differentiation and contracted in response to phenylephrine. While these TEBVs have not been studied *in vivo*, this rapid method of fabrication could significantly reduce the time to produce functional TEBVs.

Decellularized tissue contains many of the cues needed for cells to differentiate and responds dynamically after implantation, owing to cellular infiltration and imposed biomechanical loads. For example, after implantation of decellularized valves in sheep, collagen reorganized, responding to biomechanical stresses ^16^. Increased waviness of collagen corresponded to areas of greater elastin synthesis ^16^. Decellularization does cause loss and damage to some extracellular matrix proteins. To overcome this limitation, the addition of hyaluronic acid supplement enhanced adhesion in decellularized heart tissue ^17^. While decellularized tissue as thick as 1–1.5 cm can be produced, mesenchymal stem cells (MSCs) added to the decellularized constructs reached a cell density of 30 million cells/cm ^3^ but occupied only the outer 100 µm of the decellularized heart, suggesting that their growth was limited by oxygen levels. When the constructs were perfused, cells migrated as far as 400 µm into the decellularized tissue. The decellularized heart could support cardiomyocyte function as demonstrated by ESCs that exhibited beating three days after seeding ^17^.

Decellularized grafts can be modified to enhance their key functions. Immobilization of heparin to decellularized blood vessels using click chemistry reduced platelet adhesion and promoted EC attachment without altering the graft mechanical behavior ^18^. Selective attachment of biological molecules is preferable to passive adsorption in attempting to compensate for damage to the extracellular matrix during removal of cells. Rather than harvest and remove cells from blood vessels, the extracellular matrix synthesized by cultured SMCs can be used to create decellularized vessels in a tubular polyglycolic acid scaffold ^19^, thus providing a more controlled source of readily available extracellular matrix. A similar approach was used to create decellularized heart valves ^20^, which were repopulated with cells eight weeks after implantation and performed better than decellularized valves.

For valve leaflets, the need to have regional variations in cell types and material properties was achieved using decellularized valves or using injection molding ^21^ or three-dimensional bioprinting ^7^ to fabricate specific three-dimensional shapes. Since decellularized tissue can be formed into hydrogels ^22^ or electrospun, the use of different fabrication methods creates the possibility of precisely designing the tissue to be replaced.

A novel approach to generate the entire TEBV *in vivo* involves taking advantage of the foreign body response and implanting a mandrel subcutaneously around which a tubular tissue grows over a four-week period ^23^. Initially, the graft consisted of extracellular matrix and fibroblasts with a layer of M1 macrophages. After forming an end-to-end carotid anastomosis in the pig, the macrophages disappeared. After four weeks of grafting, the gene expression profile became similar to that of the carotid artery and fibroblasts adopted a contractile phenotype. The mechanical strength was very good but was less than values for actual vessels. This is a promising approach to develop engineered blood vessels, and other applications involve contracting SMCs (e.g. the bladder), which can be derived from fibroblasts involved in the foreign body response. Extending to other organ systems with specialized cells may prove difficult.

## Stem cells for tissue engineering

iPSCs offer the potential to develop engineered tissues of individual human cardiovascular disease states and avoid ethical issues associated with ESCs. iPSCs can be induced to differentiate into a large number of cell types including cardiomyocytes, SMCs ^24, 25^, and ECs ^24^. The formation of teratomas ^26^ can be reduced using non-integrating methods ^27^ and immunogenicity is low ^28^. An exciting new development has been the creation of mouse iPSCs using small molecules that activate specific transcription factors ^29^, although this approach has not yet been demonstrated with human iPSCs. A challenge with the use of iPSCs in tissue engineering is that differentiation is often limited and the resulting structures do not display a mature phenotype ^30, 31^.

Vascular cells can be obtained from iPSCs or ESCs by first activating the Wnt signaling pathway. Early activation of Wnt and β-catenin by inhibition of glycogen synthase kinase 3 (GSK3) before differentiation on surfaces with serum produces cardiomyocytes ^32^. Following Wnt pathway activation with GSK3 inhibitors, ECs can be obtained by addition of vascular endothelial growth factor (VEGF) and forskolin, while SMCs can be obtained using platelet-derived growth factor-BB (PDGF-BB) and ActivinA ^25^. Interestingly, a recent report indicated that by culturing murine iPSCs on gelatin-coated polycaprolactone nanofibrous scaffolds, Wnt/β-catenin can be transiently activated to induce differentiation towards cardiomyocytes ^33^. Combining GSK3 inhibitors with specific modification of substrate properties may lead to more robust differentiation.

One week of electrical stimulation at 0.5, 1, or 2 Hz of human ESCs or iPSCs in three-dimensional engineered tissues facilitates differentiation to cardiomyocytes by producing hypertrophy, an increase in connexin-43 gap junctions, and increased expression of hERG, the potassium channel which regulates cardiomyocyte repolarization ^34^. Some connexin-40 is expressed, indicating that rapidly conducting cells can be stimulated; however, it is not yet possible to regulate the relative expression of the various connexins though selection of a specific stimulation protocol. The stimulated cells responded to chronotropic drugs and the cells maintained synchrony to the rate of applied stimulation for two weeks after the stimulation ended.

Cardiomyocytes generated by selecting for Nkx2-5-positive cells among mouse iPSCs exhibit a number of markers found in mature cardiomyocytes, and the resting membrane potential approaches physiological levels ^35^. Three-dimensional engineered tissues produced aligned cardiomyocytes that exhibited adherens and gap junctions, although the electrophysiological responses were similar to those exhibited by fetal cardiomyocytes ^35^.

Human ESCs in three-dimensional patches showed extensive maturation and exhibited β-adrenergic responses in the physiological range ^6^. Engineered cardiac tissue derived from human ESCs integrated into damaged mouse myocardium and formed a vasculature connected to the host blood supply after 28 days but did not improve heart function owing to extensive cell loss ^36^. Alternatively, partial reprogramming of cardiac fibroblasts can be done using viral transfection of transcription factors, a cocktail of small molecules or microRNAs that activate key transcription factors (e.g. Mef2c, myocardin, and serum response factor). These approaches have yielded some success in producing spontaneously contracting cells, although the frequency of these cells among the population is low ^37^.

Culturing human cardiac myocytes derived from iPSCs on polydimethylsiloxane (PDMS) membranes coated with Matrigel for one week led to significant maturation of the cardiac cells in which the action potential upstroke velocity increased and conduction velocities were twice the value found when the cells were grown on Matrigel-coated glass coverslips ^38^, although this value was still about 57% of the *in vivo* value. This increased maturation was due to a substantial increase in increased inward rectifier potassium and sodium inward current densities, elevated connexin-43 protein expression, hypertrophy of the cardiomyocytes, and increased cardiac troponin β _1_ integrin and focal adhesion kinase. The elastic modulus of the PDMS is approximately 4 MPa, much lower than the modulus of glass (~50 GPa), and the PDMS modulus is much greater than the modulus of cardiac tissue (0.1 MPa) ^39^, suggesting that substrates with lower elastic modulus might enhance differentiation further. Modulating the substrate stiffness together with mechanical loading and electrical stimulation, which promote physiological force-frequency and force-length relations ^40^, could produce cardiomyocytes with *in vivo* electrical and mechanical properties.

The use of small molecules to differentiate iPSCs has been used to create highly differentiated ECs that model the high transport resistance of brain ECs ^41^. A number of cardiac disease models have been generated using iPSC technology and could replicate the response to cardiotoxic drugs using cells from various individuals ^42^. The technology can also be used to assess the adaptive response to dilated cardiomyopathy. For example, cardiomyocytes derived from iPSCs of healthy individuals using small molecules exhibited many of the molecules involved in β-adrenergic signaling and isoproterenol treatment induced inotropic and chronotropic regulation of contractile function ^43^. However, cardiomyocytes derived from iPSCs of individuals with dilated cardiomyopathy exhibited abnormal sarcomere structure and deficits in contractile force, calcium handling, and beat frequency after treatment with isoproterenol that was traced to overexpression of phosphodiesterases 2 and 3a ^43^.

TEBVs fabricated with SMCs derived from murine ^44^ and human ^45^ iPSCs maintained their differentiated phenotype after subcutaneous implantation for two weeks. Contractile TEBVs with SMCs differentiated from iPSCs developed from human foreskin fibroblasts and MSCs demonstrated intermediate and late SMC proteins ^46^. While TEBVs derived from karyotypically normal human iPSC clones function well and express mid-differentiation markers SM-22α and calponin and secreted extracellular matrix, those derived from karyotypically abnormal clones exhibit senescence, shortened telomeres, and calcification ^4^.

Human blood-derived ECs can be reprogrammed to SMCs by activating myocardin using a lentivirus system ^47^. Functional TEBVs were produced with these cells that exhibited flow-mediated vasodilation and vasoconstriction in the presence of 1 µM phenylephrine ^47^. While the vasoactivity was somewhat less than that of primary cells ^5^, the results do show that TEBVs can be recreated with cells from a single donor.

## Vascularization

The density and thickness of engineered tissues is limited by the transport of nutrients to the cells. Oxygen is often the limiting nutrient, since it is consumed at the highest rate and is critical for producing the energy needed for normal cell function. *In vivo*, capillary distances range from 15–50 µm depending on the cell density and the metabolic demands ^48^. *In vitro*, cell densities are lower, but uniform cell densities can be achieved only for thicknesses of about 100 µm owing to consumption of oxygen in the engineered tissue. Perfusion can lead to somewhat thicker tissues. However, without its own microvascular network that could integrate with the host network after implantation, only thin tissue-engineered constructs can remain viable after implantation.

While addition of VEGF can initiate the formation of new capillaries or branches from existing capillaries *in vitro*, the resulting structures are unstable and last at most a few days. New vessel formation involves several discrete stages. Initially, exogenous VEGF causes the release of matrix metalloproteinases, which degrade the extracellular matrix, enabling migration of the newly forming vessel buds. The newly forming vessel secretes growth factors to recruit mural cells, such as fibroblasts, pericytes, or SMCs, which interact with the newly formed microvessels, stabilizing them.

Although MSCs can stabilize EC networks *in vitro* and exhibit pericyte-like behavior ^49^, the heterogeneity of MSCs from various sources or by different isolation methods leads to variable responses ^50^. The cells must be characterized and tested for their ability to stabilize networks when developing a system to create microvascular networks. iPSCs could provide a ready source of pericytes ^51^, although SMCs or fibroblasts derived from iPSCs may be suitable.

A potentially useful model system to identify conditions that promote vascularization of tissue-engineered systems involves creating microvascular networks in a synthetic extracellular matrix hydrogel. The hydrogel contains matrix metalloproteinase degradation sites and peptide sequences of extracellular matrix proteins to elicit specific cell binding ^52^. Photopolymerization of polyethylene glycol gels enables straightforward incorporation of cells, and the networks are robust and sensitive to perfusion in the extracellular space ^52^. The direction of flow is very critical for improving mass transfer and enabling microvessels to stabilize ^53^. The extracellular matrix peptide sequences provided influence the extent of network formation, with addition of cell binding sequences from both fibronectin (RGD) and laminin (YIGSR) producing the most robust network formation in the hydrogel ^54^. Adding macrophages enhanced new vessel formation in synthetic hydrogels, consistent with their role *in vivo* ^55^. Other factors to enhance microvessel network formation in hydrogels involve regulating growth factor delivery ^56^ and a hypoxic environment ^57^.

Several approaches have been taken to incorporate vascular networks into tissue-engineered constructs for implantation. When ECs were added with MSCs to decellularized heart tissue, vascular networks formed and enabled cell growth further into the construct than could be accomplished with MSCs alone ^17^. The resulting network may have facilitated more effective fluid and nutrient transport throughout the decellularized tissue.

EC cords show promise as a method to create functional microvascular networks in engineered constructs ^58, 59^. The cords are formed by mixing ECs and mural cells in collagen. After shrinkage by 50% in diameter over four hours, the cords are encased in fibrin and integrated into the tissue-engineered construct ^58^. After implantation of cords into mice, capillaries formed within seven days and matured by 14 days. Red cells were observed in the lumen and an EC monolayer formed, defining the capillary border ^58^. The capillaries involved both donor and host ECs. When the EC and MSC cords were added with hepatic construct, they improved key hepatocyte functions ^58^. Cord diameters of 25, 75, and 250 µm all produced functional capillary networks, although smaller cords produced a higher density of vessels and the larger cords led to more dispersed vessels ^59^. Mural cells were not necessary to form functioning capillaries after implantation, possibly owing to the involvement of host mural cells ^59^. This approach can be used to control the density and geometry of the microvascular network, two properties that vary based on demand and function of the tissue.

## Microphysiological systems

High-throughput screens for function or to test drugs are being developed by integrating tissue engineering, microfluidics, and advanced methods of sensing. The National Center for Advancing Translational Science (NCATS) at NIH and the Defense Advanced Research Programs Association have led an effort to advance individual microphysiological systems and examine interactions among different organ systems. Microphysiological systems have been developed for the heart ^34, 60^, blood vessels ^5^, microcirculation ^53^, kidney ^61^, gut ^62^, lung ^63^, liver ^64^, skeletal muscle ^65^, and female reproductive tract ^66^. The small size of the systems reduces or eliminates mass transfer limitations, and function can be monitored with sensors or reporter systems. These systems have been developed using a combination of primary human cells and iPSCs. iPSCs provide the ability to create patient-specific cardiovascular disease models owing to their ability to maintain the disease phenotype post-differentiation ^67, 68^. Gene editing makes feasible isogenic controls for *in vitro* studies ^69^.

Microphysiological systems based on the cardiovascular system reproduce key functions and known drug responses. Human endothelialized TEBVs with inner diameters of 500–800 µm exhibit a dose-dependent contraction in response to phenylephrine and a dose-dependent relaxation following exposure to acetylcholine over five weeks in culture ^5^. The TEBVs elicited reversible activation to acute inflammatory stimulation by TNF-α, which was blocked by pre-treating the TEBVs with statins ^5^ and consistent with the pleotropic effect that statins exert on ECs ^70^.

Several different microphysiological systems have been developed to model cardiac function. In one, cardiomyocytes are grown on poly(N-isopropylacrylamide) (PIPAAm) in a microfluidic chamber ^71^. Forces exerted by contracting cardiomyocytes are determined from deformation of PIPAAm. As many as 28 PIPAAm cantilevers can be incorporated in one chip, and the fluidics enable easy exchange after drug or agonist exposure. This system was used to study Barth syndrome, an X-linked mutation of an acyltransferase essential for modification of cardiolipin. Individuals with this syndrome die within a year of birth due to heart failure and/or infection. This cardiac microphysiological system showed reduced contractile stresses by cardiomyocytes derived from Barth syndrome iPSCs ^72^. Contractile stresses returned to normal for cells treated with a modified RNA that corrected the mutation and was improved after treatment with linoleic acid ^72^, suggesting a novel treatment. An alternative approach to quantify contraction involves measuring strains using digital image correlation software to analyze the deformation of the engineered muscle ^34^. To convert to stress, the stress-strain behavior of the muscle is needed.

Another system provided short transport distances and confined cardiomyocytes at high cell density in a microfluidic chamber ^60^. The confinement barrier mimics the diffusive resistance of an endothelial monolayer but lacks the biochemical signals that arise from EC-cardiomyocyte interactions. Aligned and synchronously beating human cardiomyocytes derived from iPSCs were produced over a period of seven days. Cardiac cell motion was analyzed using custom software and was found to accurately represent cardiac cell responses to calcium channel and hERG blockers and β-adrenergic agonists and antagonists. The system can easily integrate the measurement of reporter fluorescence assays and analysis of the media after perfusion ^60^.

## Summary and future directions

New technologies to promote cell differentiation and vascularize engineered constructs address key challenges in making viable engineered tissues that can be implanted. At the same time, decellularized tissues, either derived from organs and tissues or fabricated in the lab, make available an alternative approach to tissue engineering in which the implanted matrix serves as a substrate to guide cell repopulation and differentiation after implantation. Both approaches have aided our understanding of the complex interactions between cells and the extracellular matrix in producing a functional tissue.
